# A study of resident duty hours and burnout in a sample of Saudi residents

**DOI:** 10.1186/s12909-018-1300-5

**Published:** 2018-08-02

**Authors:** Tahir Kamal Hameed, Emad Masuadi, Nejoud Ali Al Asmary, Faisal Ghayb Al-Anzi, Mohammed Saleh Al Dubayee

**Affiliations:** 1 King Saud bin Abdulaziz University for Health Sciences, Riyadh, Saudi Arabia; 2 Department of Pediatrics, King Abdullah Specialized Children’s Hospital, King Abdulaziz Medical City, Ministry of National Guard - Health Affairs, PO Box 22490, Riyadh, 11426 Saudi Arabia; 3 Prince Faisal Bin Bander Cancer Center, Buraidah, Saudi Arabia

**Keywords:** Resident duty hours, Post-graduate, Residency, Burnout

## Abstract

**Background:**

Work hour restrictions in residency programs have been implemented over the last several decades in Europe, USA, and Canada. To best of our knowledge, there is no study of resident duty hours in the Kingdom of Saudi Arabia. In addition, few studies have looked at the prevalence of burnout amongst Saudi residents. The present study explored resident duty hours and burnout amongst residents in Saudi Arabia.

**Methods:**

A paper-based questionnaire was designed to survey resident duty hours in Saudi Arabia and was administered along with the Maslach Burnout Inventory. The questionnaires were administered to residents in medical and surgical residency programs at King Abdulaziz Medical City-Riyadh and two hospitals in Buraidah, Qassim Province.

**Results:**

A total of 181 residents from the three hospitals participated in the survey. In terms of average number of work hours per week, 50% of all residents reported working 60–79 h while 30% reported working 80 or more hours per week. The prevalence of burnout was 81%. There was no association between higher number of working hours and the prevalence of burnout.

**Conclusion:**

This was the first study describing resident duty hours and exploring the relationship between duty hours and burnout in Saudi Arabia. Our main findings were that the majority of residents work 60 or more hours per week, and there was a very high degree of burnout amongst residents. A larger multi-centre study of resident duty hours and its effect on patient safety and resident well-being is needed to develop work hour regulations in Saudi Arabia. In addition, there is an urgent need to develop programs that address resident burnout.

## Background

Resident duty hour restrictions and modification has gained international attention over the last several decades. According to long-standing tradition, physicians in training complete long hours of on call, including working greater than 24 h shifts [1]. While being on call is an integral part of residency training, work hour restrictions were initiated to help improve both patient safety and resident well-being [2].

The original stimulus for resident duty hour reform occurred in the United States of America after the death of Libby Zion, an 18-year-old woman who died under the care of residents in New York City in 1984 [3]. Subsequently New York State adopted resident duty hour regulations in 1989 [4]. Current American guidelines follow policies set by the Accreditation Council for Graduate Medical Education (ACGME). New requirements were released in 2017 in which the maximum continuous hours of work are 24 h for all residency programs except emergency medicine which is limited to 12 h [5, 6].

The European Work Time Directive (EWTD) became law in 1998 but only in the last decade been implemented to reduce doctors’ work hours. The current guideline restricts working hours to 48 h per week [3]. In contrast to the European and American duty hour rules, there are no national duty hour restrictions in Canada. Duty hours are province dependent, and the most strict duty hour regulations has recently been implemented in the province of Quebec in which there is a 16 h limit on shifts [3].

Structured residency programs began in the Kingdom of Saudi Arabia in the early 1980s under the umbrella of the Arab Board for Medical Specialization [7]. Subsequently in 1993, the Saudi Council (Commission) for Health Specialties was established and this body gradually took over all aspects of residency programs in Saudi Arabia [8]. There are currently 24 specialties in the country with over 700 accredited training programs [9]. While resident duty hours have gained international attention, there has been no study of resident duty hours in Saudi Arabia. Duty hours in Saudi residency programs are generally determined by each individual residency program. The Saudi Commission for Health Specialties outlines some guidelines for on call for training programs; for example, for pediatrics the Program Manual states that the average calls for junior residents (R1-R2) should be seven per month while for senior residents (R3-R4) should be six per month. In addition, calls should be 24 h and the resident should leave from 12:00 pm post-call [10]. These guidelines however do not regulate the total number of work hours per week.

Burnout is very common amongst residents with a prevalence of up to 76% in some studies [11, 12]. Burnout is a syndrome characterized by a loss of enthusiasm for work (emotional exhaustion), feelings of cynicism (depersonalization) and a low sense of personal accomplishment [13]. Residents are at high risk for developing burnout likely due to multiple factors including long working hours, high stress levels and sleep deprivation [14]. There have been only an handful of studies in Saudi Arabia describing burnout amongst residents, and a recently published survey of plastic surgery residents found a high degree of burnout (71% with a high degree of burnout on the emotional exhaustion scale) [15]. With physician burnout being associated with medical errors and suboptimal patient care [11, 12], we believe that it is important to study resident duty hours and burnout amongst residents in Saudi Arabia and to determine if any relationship exists between the two. The research objectives of this study were to: 1) describe resident duty hours in residency training programs in Saudi Arabia; 2) determine the prevalence of burnout amongst residents in different specialties in two geographical regions in the kingdom; and 3) describe the relationship between residency duty hours and burnout amongst residents.

## Methods

### Survey design and administration

As part of research on duty hours, burnout, sleep and depression amongst Saudi medical and surgical postgraduate trainees, a paper-based 19-item questionnaire in the English language was designed to survey resident duty hours in Saudi Arabia. This questionnaire was designed after reviewing a published questionnaire surveying duty hours in family medicine residents [16]. Along with the duty hours questionnaire, we used the validated 22-item Maslach Burnout Inventory (MBI) to measure burnout. The MBI is recognized as the leading measure of burnout. A high degree of burnout is reflected in high scores on the emotional exhaustion (EE) and depersonalization (DP) subscales and low scores on the personal accomplishment (PA) subscale [17]. Consistent with many other studies, we defined burnout using the most common definition, high subscore on either the EE or DP subscales (EE ≥ 27 or DP ≥ 10) [17, 18].

These questionnaires were administered to residents in accredited medical and surgical residency programs in two different regions in the kingdom: King Abdulaziz Medical City-Riyadh (KAMC-R) which is under the Ministry of National Guard - Health Affairs (MNG-HA) (Riyadh province) and Maternity and Children’s Hospital Buraidah and King Fahad Specialist Hospital Buraidah (both in Qassim province). KAMC-R is a large academic health care institution in the Kingdom and questionnaires were distributed to residents in all the medical and surgical training programs. Residents in pediatrics, internal medicine, and general surgery were surveyed at the two institutions in Qassim. The questionnaires were distributed during the 2013–2014 academic year. Non-medical programs (e.g. dental) were excluded. Informed consent was obtained from all participants through an invitation letter outlining the objectives of our study. The invitation letter stated that completion of the questionnaire implies consent and that responses would remain confidential (no names were recorded on the questionnaires). As an incentive to participate, participants could voluntarily enter their names to win gift cards for a local bookstore. Ethics approval for this study was obtained from the Institutional Review Board at King Abdullah International Medical Research Center, MNG-HA, Riyadh (Ref. #: IRBC/055/12).

### Data analysis

Descriptive statistics (frequencies and percentages) were used to summarize the quantitative variables. For comparisons between residency programs, centres, and categorical variables, the chi square or Fisher’s exact test were used. Significant differences were identified at *P* < 0.05. Statistical analysis was performed with SPSS version 22 (IBM Corporation, USA).

## Results

Out of a total of 425 residents surveyed from the three hospitals, 181 residents completed the survey giving an overall response rate of 43%. One hundred and six of the respondents (59%) were female. One hundred and forty six residents (82%) were from KAMC-R, and 136 (76%) were in non-surgical residency programs. Of non-surgical residents, 96 (71%) were pediatric or internal medicine residents, whereas for surgical residents 35 (80%) were obstetrics/gynecology or general surgery residents. The average age of residents was 27.6, slightly more than one-half were married, and 30% had children. Greater than 80% of the residents were in their first to third years of training (Table 1).

**Table 1:** Baseline characteristics of Residents Completing Saudi Resident Duty Hours Survey

	N	Percent
Gender		
Male	75	41.4
Female	106	58.6
Marital status		
Married	86	48.0
Unmarried	93	52.0
Children		
No	109	69.9
Yes	47	30.1
Institution		
KAMC-R	146	81.6
Qassim^a^	33	18.4
Specialty		
Surgical	44	24.4
Non-Surgical	136	75.6
Level of training		
R1	65	36.1
R2	48	26.7
R3	37	20.6
R4	24	13.3
R5	6	3.3
Average number of working hours per week (including on-call)		
< 40	7	4.0
40–59	28	16.0
60–79	87	49.7
80–99	39	22.3
≥ 100	14	8.0

^a^Qassim: Maternity and Children’s Hospital Buraidah, and King Fahad Specialist Hospital Buraidah

In terms of average number of work hours per week (including on call hours), 87 (50%) of all residents reported working 60–79 h while 39 (22%) reported 80–89 h per week. Fourteen residents (8%) reported working equal to or more than 100 h per week. Almost one-half of residents reported “reasonable working hours” as 40–59 h per week, whereas 32% reported less than 40 h per week (Table 2). Greater than two-thirds of residents worked 24–28 continuous hours when on-call, while just over one-quarter worked 29 or more hours. More than 80% stated that 5–8 calls was the maximum number of on calls per month, while almost 10% had 9 or more calls per month.

**Table 2:** Prevalence of Burnout Characteristics Amongst Saudi Residents

Characteristic	Percentage
Personal Achievement (PA)	
Low	11.1
Medium	22.2
High	66.7
Emotional Exhaustion (EE)	
Low	12.8
Medium	25.0
High	62.2
Depersonalization (DP)	
Low	10.6
Medium	18.9
High	70.6
Burned out^a^	80.7

^a^Defined as high EE or DP

The vast majority of residents (85%) reported that their residency program does not have any policy on the maximum number of working hours per week, though about one-half did report a policy about “on-call” duration. Almost 80% of residents felt that the duration of on call is too long, and about two-thirds report excessive stress due to the length of working hours and on-calls.

Burnout subscales were stratified into mild, moderate and high (Table 3). Of all respondents, 67, 62, and 71% scored high from the PA, EE, and DP subscales respectively. The overall prevalence of burnout was 81%. Female gender and married status were significantly associated with a higher prevalence of burnout (*p* = 0.036 and 0.016, respectively). Being a female resident increases the chance of burnout by 2.21 times as compared to a male resident, while being a married resident increases the chance of burnout by 2.64 times as compared to an unmarried resident. There was no association between higher number of working hours and prevalence of burnout (*p* = 0.105).

**Table 3:** Factors Associated with Burnout Amongst Saudi Residents

Variable	Categories	Burned out (%)	OR	95% CI for OR		*P* value
				Lower	Upper	
Gender	Female	85.8	2.21	1.04	4.66	0.036
	Male^a^	73.3	1			
Marital status	Married	88.4	2.64	1.18	5.92	0.016
	Unmarried^a^	74.2	1			
Have any children?	Yes	83	1.30	0.54	3.17	0.558
	No^a^	78.9	1			
Institution	Qassim	84.8	1.45	0.52	4.07	0.480
	KAMC-R^a^	79.5	1			
Specialty	Surgical	77.3	0.77	0.34	1.75	0.527
	Non-Surgical^a^	81.6	1			
Junior vs. Senior Resident	Junior resident	81.4	1.16	0.54	2.47	0.705
	Senior resident^a^	79.1	1			

*OR* odds ratio, *CI* confidence interval

^a^reference group

## Discussion

We present the first study of resident duty hours in Saudi Arabia, and the most recent multi-centred, multi-specialty survey of burnout amongst Saudi residents. Our main findings were that the majority of residents work 60 or more hours per week, there was a very high prevalence of burnout amongst residents (81%), and there was no association between working more hours and burnout.

Approximately 50% of residents worked 60–79 h per week (including on-call hours), while more than 30% reported working more than 80 h per week. This high proportion of residents working extended hours is alarming but not surprising given there were no guidelines on the maximum number of work hours per week in the Kingdom. One of the main reasons of duty hours implementation is to improve patient safety. However there is conflicting evidence in the literature to support or refute duty hours restrictions’ effect on improving patient safety. In the systematic review published by Fletcher et al. [19] there was improvement in patient mortality after implementation of the 2003 ACGME Duty Hour Rules. In contrast, in the most recent randomized control trial of duty-hour flexibility in surgical training (the FIRST Trial), flexible, less-restrictive duty hour policies for surgical residents were associated with noninferior patient outcomes [20]. Duty hour restrictions may result in more medical errors because of increased handoffs and transitions of care [2]. While it is not clear at what number of duty hours that patient safety may be compromised, we are concerned that almost one-third of Saudi residents in our study work more 80 or more hours per week. More objective studies are needed on patient safety and outcomes with extended duty hours.

In comparison to other studies of burnout amongst Saudi residents, our study shows a higher degree of burnout than reported before in the Kingdom. The previously mentioned study of plastic surgery residents in Saudi Arabia reported that 71% of residents met a high degree of burnout in the EE subscale [15]. A burnout study that was conducted in 2005 at KAMC-R and three other hospitals found an overall burnout prevalence of 70% [21]. Why resident physicians in Saudi Arabia may experience a higher degree of burnout compared to residents across the globe is not clear. Some authors have postulated that the social, cultural and economic set-up in the Kingdom may impact the prevalence of burnout. Because of the lack of health education in the general public, Saudi patients tend to expect much more out of physicians. There is also a cultural tendency of Saudi patients and their relatives to seek advice and direct their attention at more senior doctors and to ignore junior doctors in the process. This may lead to a feeling of reduced worthiness, and might therefore increase burnout [21]. We postulate that the high degree of burnout experienced by Saudi residents is also due a lack of programs to address stress and burnout. In addition, the implementation of mentorship programs which may help address resident well-being are just now being implemented in Saudi residency training programs. We found a positive association between burnout and female sex and married status. In the only prior multicenter multispecialty study of resident burnout in the Kingdom, female residents had a higher degree of burnout on one of the subscale while marital status did not have an impact on burnout [21]. Married residents may have added responsibilities which could add to burnout. However a systematic review of burnout in residency training found conflicting results of the effect of gender and marital status on burnout [22].

Duty hour standards have been implemented internationally in an effort to improve resident well-being. We did not observe any association between longer working hours and burnout. While some older studies did show a higher burnout prevalence as working hours increased [23, 24], more recent studies have shown conflicting results. In a national survey of burnout among US general surgery residents, Elmore et al. [25] found a higher burnout on EE and DP was associated with greater work hours per week. In contrast, amongst pediatric residents, work hours in the past week were not a significant predictor of burnout [14]. Though intuitively longer working hours should be associated with higher burnout, we believe one consequence of reducing duty hours is “work compression” which increased perceived resident workload and may in fact contribute to increased burnout [26]. A recent study of residents in Nigeria found that heavy workload was significantly associated with each of the three dimensions of burnout [27]. We also believe that there are many risk factors for burnout like sleep and depression, and work hours is only one factor.

There are several strengths of our study. Our research is the first study in the region to survey resident duty hours. In addition, we surveyed residents in three hospitals in two geographically different locations. Furthermore, our study is the most recent multi-centre, multi-specialty survey confirming that burnout is very common amongst Saudi resident physicians. The main limitation of this study is the relatively small sample size making it difficult to generalize our findings. Moreover, the residents completed the survey at different times during the academic year and this may have affected the rate of burnout. Another limitation of our study is that it was conducted approximately 4 years ago. However there have been no major changes in graduate medical education in Saudi Arabia nor any formal duty hour regulations during this time period that may have influenced the findings of our research.

## Conclusions

Residents in Saudi Arabia report working long hours and have a high degree of burnout. There has been recent work on developing national duty hour regulations in the Kingdom but the regulations are not publically available at this time. While there is no clear association between long duty hours and resident burnout, there is a need for developing national duty hour regulations in Saudi Arabia to protect residents’ rights and maintain patient safety. As stated by Shanafelt, Dyrbye and West [28], although the problem of physician burnout has been widely recognized, there is less information on how to address this important problem. Moving forward, there is a need for a national study on burnout amongst residents in Saudi Arabia and an urgent need to develop programs that address resident burnout. Resident physicians must look after their own well-being first in order to provide optimal care for others.

## Abbreviations

ACGME: Accreditation Council for Graduate Medical Education
DP: Depersonalization
EE: Emotional exhaustion
KAMC-R: King Abdulaziz Medical City-Riyadh
MBI: Maslach Burnout Inventory
MNG-HA: Ministry of National Guard – Health Affairs
PA: Personal accomplishment
